# Structure-Based Understanding of Binding Affinity and Mode of Estrogen Receptor α Agonists and Antagonists

**DOI:** 10.1371/journal.pone.0169607

**Published:** 2017-01-06

**Authors:** Sehan Lee, Mace G. Barron

**Affiliations:** U.S. Environmental Protection Agency, Gulf Ecology Division, Gulf Breeze, FL, United States of America; University of Michigan, UNITED STATES

## Abstract

The flexible hydrophobic ligand binding pocket (LBP) of estrogen receptor α (ERα) allows the binding of a wide variety of endocrine disruptors. Upon ligand binding, the LBP reshapes around the contours of the ligand and stabilizes the complex by complementary hydrophobic interactions and specific hydrogen bonds with the ligand. Here we present a framework for quantitative analysis of the steric and electronic features of the human ERα-ligand complex using three dimensional (3D) protein-ligand interaction description combined with 3D-QSAR approach. An empirical hydrophobicity density field is applied to account for hydrophobic contacts of ligand within the LBP. The obtained 3D-QSAR model revealed that hydrophobic contacts primarily determine binding affinity and govern binding mode with hydrogen bonds. Several residues of the LBP appear to be quite flexible and adopt a spectrum of conformations in various ERα-ligand complexes, in particular His524. The 3D-QSAR was combined with molecular docking based on three receptor conformations to accommodate receptor flexibility. The model indicates that the dynamic character of the LBP allows accommodation and stable binding of structurally diverse ligands, and proper representation of the protein flexibility is critical for reasonable description of binding of the ligands. Our results provide a quantitative and mechanistic understanding of binding affinity and mode of *ER*α agonists and antagonists that may be applicable to other nuclear receptors.

## Introduction

Endocrine disrupting chemicals (EDCs) are exogenous agents that interfere with endocrine systems and produce a range of reproductive, developmental, and metabolic dysfunctions in both humans and wildlife [1–4]. EDCs consist of highly heterogeneous natural and synthetic compounds, and many of these environmental contaminants act via nuclear receptors (NRs). NRs are ligand-modulated transcription factors that regulate expression of genes controlling a wide range of developmental and physiological processes. NR ligand binding domains (LBDs) typically contain ten to twelve α helices that create a flexible hydrophobic ligand binding pocket (LBP) able to interact with a large diversity of molecular structures [5, 6]. While the overall architectures of NR LBDs are similar, the LBPs are sufficiently unique in amino acid compositions to ensure specificity for endogenous ligands [7].

Estrogen receptor α (ERα) is one of the most extensively studied NR targets related to the effects of EDCs [8, 9]. The ERα LBD consists of eleven helices (H1, H3-H12) arranged in three layers to form a large hydrophobic pocket that allows the binding of a wide variety of non-steroidal compounds through hydrophobic interactions. Extensive crystallographic studies of human ERα (hERα) in complex with various agonists and antagonists [10–13] have provided insights into the essential structural features necessary for high ligand binding affinity. These include: 1) hydroxyl groups that serve to anchor the ligand in the ERα LBP via hydrogen bonds with polar amino acids including Thr347, Glu353, Arg394, and His524 and 2) a conserved hydrophobic core structure typically composed of a phenyl, analogous to the A-ring of 17β-estradiol (E2), and a substituent that occupies the remaining large volume of the LBP equivalent to C- and D-rings. The LBD also contains the activation function 2 (AF2) whose action is dependent on the presence of a bound ligand [14]. When the ERα LBD is bound to agonists, H12 is positioned over the LBP and forms AF2 together with H3, H4 and H5 for the recruitment of coactivators. However, when an antagonist binds to the ERα LBD, a tertiary amine at the end of the antagonist side chain forms a salt-bridge with Asp351 and forces H12 reposition over the coactivator binding groove, preventing the coactivator recruitment.

Despite the crucial insight provided by experimental [15–17] and theoretical [18, 19] investigations of NR binding, the quantitative contribution of specific protein-ligand interactions to binding affinity and mode are still largely uncharacterized, in particular for the hydrophobic contacts that dominate the LBP. Our recent studies [20, 21] have shown that a 3D-QSAR approach combining molecular docking, structure-based pharmacophore, and 3D-fingerprint can provide a quantitative understanding of how structurally and chemically diverse compounds that have different binding modes and mechanisms of action can interact with acetylcholinesterase and inhibit its activity. In the current study, we report a quantitative analysis of the role of individual ERα-ligand interaction features using the 3D-QSAR approach and a novel hydrophobicity density field descriptor, followed by prediction of binding affinity and mode of a wide range of compounds to validate our analysis. This work represents an advancement over previous ERα modeling based on QSAR, molecular dynamics simulations, pharmacophores, and molecular docking [22–26] because of quantitation of hydrophobic contributions to the binding affinity and mode of ERα agonists and antagonists. Additionally, the mechanistic understanding of how the LBP allows accommodation and stable binding of structurally diverse ligands through interaction features and protein flexibility has implications for modeling other nuclear receptors.

## Materials and Methods

### Data preparation

Chemical structures and *in vitro* estrogenic activities expressed as relative binding affinity (RBA = (E2 IC_50_/Competitor IC_50_)×100) were obtained from EDKB [27], ChEMBL [28], and other literatures [29, 30]. Chemical structures were protonated and energy minimized with MMFF94x using MOE (Chemical Computing Group). 73 X-ray crystal structures of hERα LBD in complex with 61 agonists and antagonists were downloaded from Protein Data Bank [31] for structure-based pharmacophore modeling. RBA values of 31 out of the 61 ligands were available and used for the QSAR model development. RBA values of 111 ligands from EDKB, excluding extremely flexible compounds (the number of rotatable bonds > 10), were used for external validation of the model. Ligand structures are given in S1 and S2 Files.

### 3D-Fingerprint descriptor

Selective binding of a ligand to a specific protein is determined by structural and energetic recognition of the ligand and the macromolecule. Key protein-ligand interaction features were identified using a structure-based pharmacophore approach, beginning with a search for common steric and electronic features in the 73 X-ray crystal structures of hERα LBD. Protein-ligand complex structures from x-ray crystallography and molecular docking were mapped onto the developed pharmacophore and transformed into a 3D-fingerprint as a descriptor encoding protein-ligand interactions. Each bit of the fingerprint represents a pharmacophore feature.

### 3D-QSAR development

Multiple linear regression combined with genetic algorithm (GA-MLR) was carried out using the RapidMiner5.2 tool (http://rapid-i.com) to select important interaction features and analyze their quantitative contributions in ERα binding. The model was validated by leave-one-out cross-validation.

### Hydrophobicity density field

**Fig 1:**
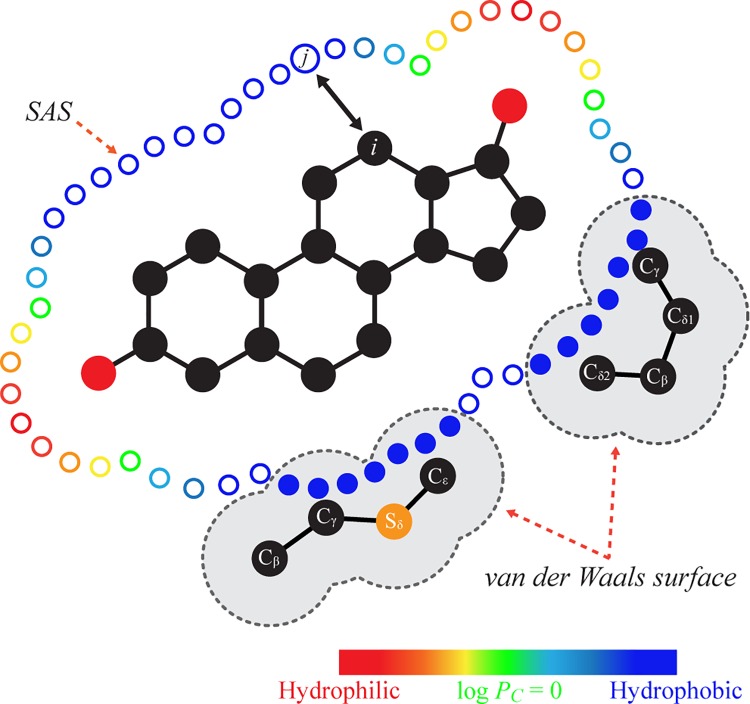
Description of hydrophobic interactions on the contact surface. Hydrophobic and hydrophilic grid points are represented by blue and red circles, respectively, on the SAS of the ligand. Hydrophobic grid points within the *van der Waals* surface of hydrophobic residues are marked by filled blue circles.

To measure the hydrophobic interactions on the contact surface log *P*_*C*_, an empirical hydrophobicity density field model was applied (Fig 1). A ligand was described in terms of a grid on its solvent accessible surface (SAS), and hydrophobicity density at a grid point (log *P*_*j*_) was calculated using Generalized-Solvation Free Energy Density (G-SFED) model [32]:

$$\log P_{j}=C_{1}\left|\sum_{i=1}^{N_{A}}\frac{q_{i}}{r_{ij}^{2}}\right|+C_{2}\sum_{i=1}^{N_{A}}\frac{q_{i}^{2}}{r_{ij}^{3}}+C_{3}\sum_{i=1}^{N_{A}}\frac{\alpha_{i}}{r_{ij}^{3}}+C_{4}\sum_{i=1}^{N_{A}}\frac{\alpha_{i}}{r_{ij}^{6}}+C_{cav}$$ (1)

where *N*_*A*_ is the number of atoms of the ligand, *r*_*ij*_ is the distance between the *i*th atom and the *j*th grid point, *q*_*i*_ is the net atomic charge [33], and *α*_*i*_ is the effective atomic polarizability [34]. The coefficients, *C*_1_, *C*_2_, *C*_3_, *C*_4_, and *C*_*cav*_, represent the solvent effect on various solvent-solute interactions and were -8.85∙10^−2^, -1.45∙10^−1^, 1.57∙10^−2^, -4.88∙10^−1^, 2.34∙10^−3^, respectively. The log *P*_*C*_ was obtained by integrating hydrophobic grid points (log *P*_*j*_ > 0) on the contact surface:

$$\log\;P_{C}=\sum_{k}^{N_{R}}\sum_{j}^{C_{k}}\log\;P_{j}$$ (2)

where *N*_*R*_ is the number of hydrophobic residues in the LBP (S1 Table), and *C*_*k*_ is a set of hydrophobic grid points within the *van der Waals* surface [35] of the *k*th hydrophobic residue.

### Molecular docking and bioactive conformation selection

Molecular docking simulations were conducted with AutoDock Vina [36] using default parameters. For more thorough search of conformational space, 10 independent docking simulations were performed on each protein-ligand complex. Among a large number of docked conformations generated by the repeated docking simulations, the conformations observed three or more times (RMSD < 1.0 Å) were selected as candidates of the bioactive conformation to maximize the reproducibility of the results and reduce false positives of low possibility. The selected candidate conformations of a ligand were scored by RBA estimated with the QSAR model, and the best-scored conformation was selected as a bioactive conformation of the ligand [20].

## Results

### 3D-QSAR for understanding binding affinity and mode

A 3D-QSAR model was developed to quantitatively analyze the binding affinity and mode of structurally diverse ERα agonists and antagonists. The developed structure-based pharmacophore model consisted of nine candidate features including 1) a salt-bridge or acid-acid interaction [37] with Asp351, 2) five hydrogen bonds with Leu346, Thr347, Glu353, Arg394, and His524, 3) a T-shaped π-stacking with Phe404, 4) the number of internal hydrogen bonds in ligand, and 5) hydrophobic contact (log *P*_*C*_) with hydrophobic residues in the LBP. Geometrical constraints for the features are summarized in S1 Fig.

**Fig 2:**
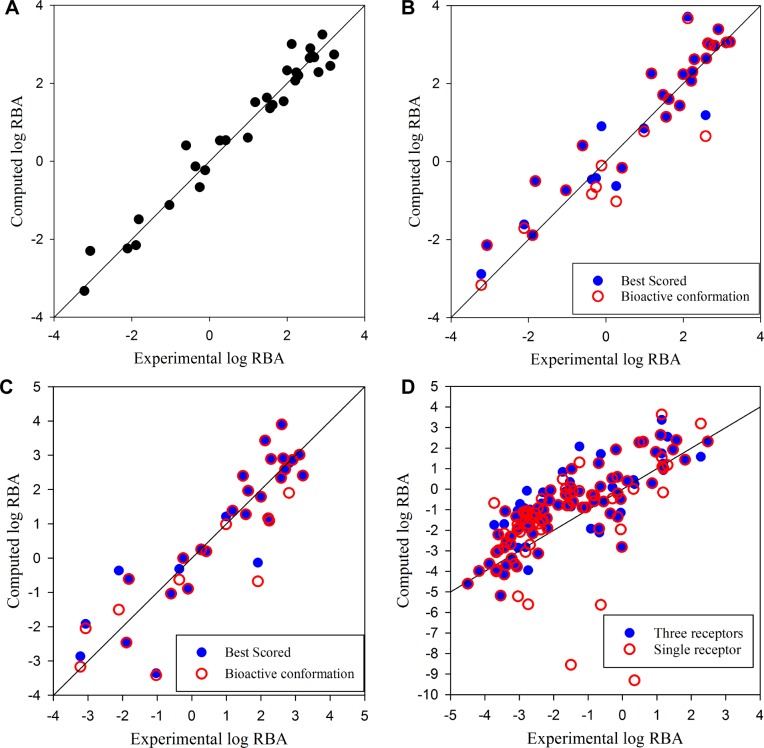
Scatter plots of log RBA calculated for 31 training ligands (A, B, and C) and 111 external test ligands (D). Protein-ligand complex structures were obtained from crystal structures (A), self-docking (B), cross-docking (C), and single or three receptor structures-based docking (D).

S2 Table contains the 3D-fingerprints and predicted RBAs for the 73 crystal structures together with the available 31 experimental RBA values. The optimal 3D-QSAR model had six bits (four binary bits for a salt-bridge (FP1) and three hydrogen bonds (FPs2-4), an integer bit for the number of internal hydrogen bonds in the ligand (FP5), and a continuous bit for log *P*_*C*_ (FP6)). The model exhibited significant self-consistency (R^2^ = 0.96, Fig 2*A*) as well as high internal predictive ability (Q^2^ = 0.93). The hydrophobic interaction was sensitive to the geometry of the protein-ligand complex. The log *P*_*C*_ values calculated for crystal structures bound to a ligand differed up to 0.27, which corresponded to an approximately 11-fold difference in RBA (ligand 3 in S2 Table). The largest RBA residual was 10-fold (ligand 29), which is within the uncertainty range of the crystal structures. A summary of the developed pharmacophore, fingerprint, and 3D-QSAR models is provided in Table 1.

**Table 1:** Summary of pharmacophore, fingerprint, and QSAR model parameters.

Pharmacophore		Fingerprint Index	QSAR Coefficient
Type	Amino acid		
Salt-bridge	Asp351	FP1	0.923
Hydrogen bond	Glu353	FP2	2.209
Hydrogen bond	His524	FP3	1.689
Hydrogen bond	Thr347	FP4	1.487
Internal hydrogen bond of ligand	NA^*a*^	FP5	0.614
log *P*_*C*_	Hydrophobic residues^*b*^	FP6	3.861
Intercept	NA	NA	-9.341

^*a*^NA, not applicable

^*b*^See S1 Table

### Binding mode prediction combined with molecular docking

A ligand bound to a receptor adopts a conformation that can form stabilizing protein-ligand interactions described by binding affinity. To validate how well our model can explain the ERα binding of structurally diverse ligands, we predicted the binding mode of the 73 crystal structures based on the 3D-QSAR model combined with molecular docking. Each of the co-crystallized ligands was docked into the protein structure from which it was extracted (self-docking) to generate possible conformations of the ligand in the LBP. The 3D-QSAR showed highly accurate binding mode predictions with the 48 bioactive conformations of 40 ligands (66 percent) ranked in the first position and only three bioactive conformations of ligands 7, 34, and 60 ranked in the fourth or lower position (Table 2). The misidentifications were primarily attributed to the inaccuracy in docked conformations. The self-docking experiments generated bioactive conformations within 2 RMSD to the crystal structures for all tested ligands. However, the deviation at the large binding pocket around the C and D-rings of E2 led to the misidentification of the hydrogen bond with His524 and resulted in greater than 10-fold over or under estimations for ligands 22, 34, and 60. Furthermore, estimation of log *P*_*C*_ was impaired by steric collisions, especially around the narrow A-ring region, due to the merging non-polar hydrogen atoms to heavy atoms [36]. Although the 22 bioactive conformations of 21 ligands were ranked in the second or third position due to these steric collisions, the difference of estimated RBAs between the best scored and bioactive conformations was within 1 order of magnitude (Fig 2*B*). Therefore, r^2^ values for the best-scored and bioactive conformations were comparable for the 31 training crystal structures (0.89 and 0.87, respectively), which indicates that the model can provide accurate RBA prediction given the correct receptor structures.

**Table 2:** Accuracy as rank of binding mode prediction based on the 3D-QSAR combined with self-docking or cross-docking.

Docking Method	Rank^*a*^				
	1st	2nd	3rd	≥ 4th	NI^*b*^
Self-docking	48	18	4	3	0
Cross-docking	42	12	8	5	6

^*a*^Rank: relative position of the bioactive conformation within docked conformations sorted by predicted RBA

^*b*^The number of ligands for which molecular docking could not generate the bioactive conformations

### Prediction of binding affinity of external set of structurally diverse compounds

**Fig 3:**
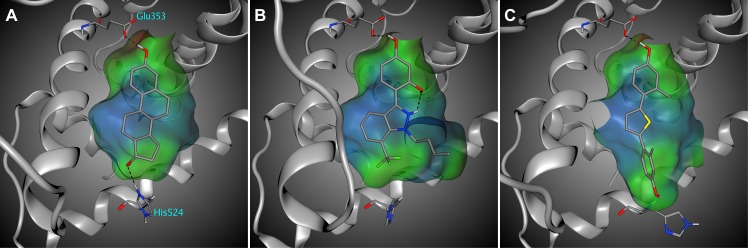
Classification of His524 conformations (PDB IDs: 2YJA, 4IVY, and 4IWC). Closed (A) and moved back (B) conformations which stabilize protein-ligand complex through hydrogen bond and hydrophobic interaction, respectively. Open conformation (C) that provides an expanded binding pocket for ligands longer than 13 Å. Ligands are colored in orange with hydrogen, oxygen, nitrogen, sulfur, and fluorine atoms in white, red, blue, yellow, and green, respectively.

Preliminary docking experiments using the 73 crystal structures were performed to evaluate possible performance loss in a practical application where a crystal structure of ERα in complex with a compound is unavailable. A ligand extracted from a crystal structure was docked into a receptor structure from a complex containing a different ligand with the highest molecular similarity (cross-docking). The RDKit topological fingerprint (www.rdkit.org/) based Tanimoto coefficient was used to compute the similarity and ranged from 0.46 to 0.99 with average of 0.78. The 3D-QSAR approach based on the cross-docking showed reduced but still accurate performance, where 42 bioactive conformations of 33 ligands were ranked at the first position (Table 2), and r^2^ values for the best-scored and bioactive conformations were 0.79 and 0.80 (Fig 2*C*), respectively. The cross-docking could not find bioactive conformations of ligands 23, 24, 35, 40, 54, and 56 due to the steric hindrance with flexible residues, especially His524. His524 adopts a spectrum of conformations depending on the steric and electronic properties of the ligand. When ERα is bound to a ligand in which a phenol group and a hydroxyl group are 10–12 Å apart from each other, His524 adapts a conformation that can form a hydrogen bond with the hydroxyl group (“closed” conformation, Fig 3*A*). If a hydrophobic group is present instead of the hydroxyl group, His524 adapts a conformation that can form hydrophobic interactions or reduce steric collisions (“moved back” conformation, Fig 3*B*). The other conformation is the “open” arrangement that provides an expanded binding pocket for ligands longer than 13 Å (Fig 3*C*).

The 3D-QSAR approach was validated using 111 structurally diverse ERα binders including phytoestrogens, bisphenols, steroids, pesticides, DDTs. The receptor structures from the 73 crystal structures were grouped into three receptor structure classes (closed, moved back, and open) based on the conformation of His524 (S2 Table). Each ERα binder was docked into three ERα structures from each receptor structure class selected by Tanimoto coefficient between a co-crystallized ligand and the binder. Tanimoto coefficients ranged from 0.36 to 1.00 with average of 0.68, which was lower than the cross-docking results.

Among a large number of protein-ligand complex structures generated by docking experiments, only those satisfying the geometric relationship between the docked ligand and His524 were considered (S2 Fig.). To reduce the error from steric collisions with the non-polar hydrogens, only the docked conformations with proper arrangement of the phenyl group at the A-ring region (S1*D* Fig) were considered. Inclusion of conformational changes of His524 allowed the generation of reasonable docked conformations of compounds within the LBP, in contrast to docking experiments using only a single receptor structure. The 3D-QSAR approach based on single and three receptor structures had r^2^ values of 0.48 and 0.66 for the external test set (Fig 2*D*), respectively.

## Discussion

It is challenging to establish a complete and confident understanding of ERα, mainly due to its promiscuous LBP. Although the “pincer-like” arrangement around the A-ring imposes an absolute requirement on ligands to contain an aromatic ring aligned vertically to the phenyl ring of Phe404, the remaining large flexible hydrophobic binding pocket can accommodate a broad range of hydrophobic groups through hydrogen bonds and hydrophobic interactions [10]. In this study, we performed quantitative analysis of ERα binding affinity and mode by combining effective 3D protein-ligand interaction descriptions with 3D-QSAR approach.

Binding affinity was expressed as a linear combination of interaction features.

$$\log\;\text{RBA}=\sum_{i=1}^{6}c_{i}{\text{FP}}_{i}+C$$ (3)

The product of a bit in the 3D-fingerprint, FP_*i*_, and its regression coefficient, *c*_*i*_, represents the independent contributions of each interaction feature to binding affinity. The intercept *C* is the binding affinity without any protein-ligand interactions, which is approximately 10^−9^ RBA. A phenolic group could act as a hydrogen bond donor and acceptor for Glu353 and Arg394, respectively. However, the 3D-QSAR results indicated that whereas the hydrogen bond donation (FP2) is a significant stabilizing interaction with ERα (162-fold increase in RBA), the hydrogen bond acceptance is negligible. This is consistent with the experimental evidence that the phenolic group of E2 probably acts primarily as a hydrogen bond donor [38]. A hydroxyl group that is about 11 Å apart from the phenolic group could form a hydrogen bond with His524 (FP3) and increase binding affinity approximately 50-fold. A hydrogen bond with Thr347 (FP4) was observed only in three diphenylmethane derivatives [12, 14] and contributed to a 30-fold increase in RBA. Another possible hydrogen bond feature that interacts with the amide carbonyl of Leu346 was observed in WAY-derivative ligands 46, 49, 50, and 52 [13], but was not considered in this study because of lack of experimental binding affinity data for the ligands.

Binding affinity of the agonists and antagonists was markedly dependent on the nature of the core structure of the ligand. The hydrophobic core extensively interacted with hydrophobic residues in the LBP, and hydrophobic substituents having different size and shape at various positions could enhance binding affinity. Although there are no theoretical or experimental studies for estimating the quantitative contribution from the hydrophobic contacts, the measurement of relative gas-phase stability for hERα-ligand complexes [15, 17] confirmed the prominent role of the hydrophobic contacts for stabilizing the complexes in solution. In this study, the calculated log *P*_*C*_ values for the 31 training crystal structures ranged from 0.80 (ligand 28 in S2 Table) to 2.44 (ligand 16) corresponding with from 3.09 to 9.42 orders of magnitude in RBA. This high variance of log *P*_*C*_ accounts for the 10^6^-fold difference in RBA between ligands 16 and 31, which form a hydrogen bond with Glu353. Each internal hydrogen bond enhancing the molecular hydrophobicity contributed to an approximately 4-fold increase in RBA and accounted for the comparable or higher RBA of flavones, isoflavones, and flavaonones with a hydroxyl group participating in an internal hydrogen bond [16].

**Fig 4:**
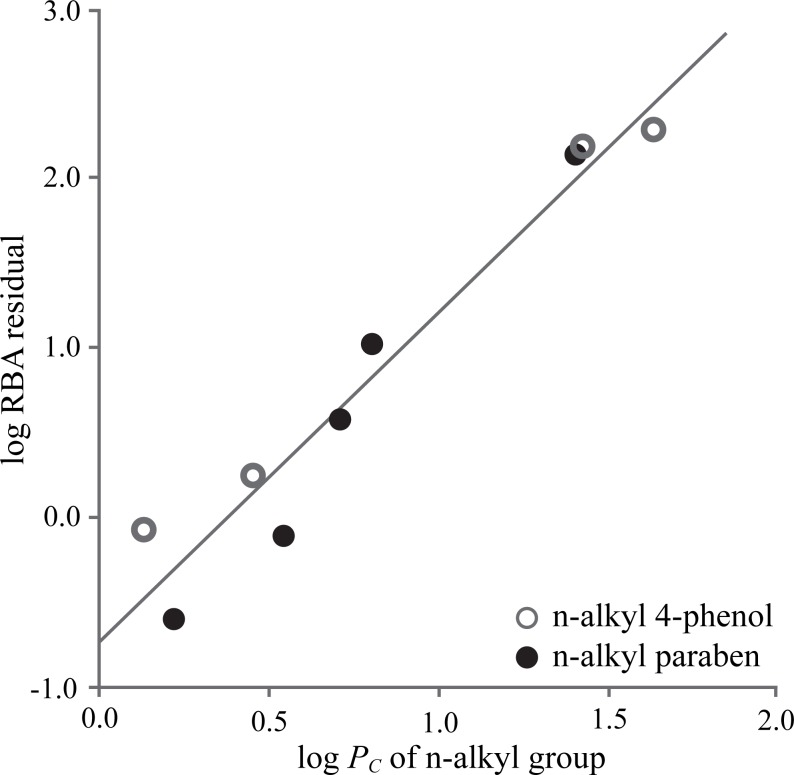
Hydrophobic contacts (log *P*_*C*_) of n-alkyl group vs log RBA residual of n-alkyl 4-phenol (gray circles) and n-alkyl paraben (black filled circles).

Hydrophobic contact within the hERα LBP is a major determinant of binding affinity, but nonspecific interaction. Without hydrogen bond or steric hindrance to reduce degree of rotational and translational freedom, small and/or flexible ligands can adapt alternate conformations in the LBP, which destabilizes the hydrophobic contacts and reduces RBA. Superimposition of subunits A and B from ERα homodimer crystal structure in complex with partial agonist butylparaben [14] showed that the flexible butyl group adopts different conformations with an RMSD of 1.45 Å. Since this destabilization was not reflected in the log *P*_*C*_ calculation, RBA of ERα-butylparaben crystal structure was overestimated by 6-fold. The external validation results of n-alkyl 4-phenols and n-alkyl parabens (1 ≤ n ≤ 9) based on docked conformations clearly showed that the flexible n-alkyl groups form unstable hydrophobic contacts, which results in a residual that is proportional to log *P*_*C*_ of the n-alkyl groups (Fig 4). This observation is consistent with our 3D-QSAR results for acetylcholinesterase inhibition potency, in which the contribution from hydrophobic interaction with a free aliphatic leaving group is much smaller than that with aliphatic side chain stabilized by covalent bond with serine residue [21].

The bulky hydrophobic side chain of typical ERα antagonists makes extensive hydrophobic contacts with helices H3, H5/6, and H11 [10]. A tertiary amine at the end of the side chain, which is protonated and forms a salt-bridge with carboxylate of Asp351, generates strong electric field reducing hydrophobicity of the SAS around the tertiary amine. Although the salt-bridge accounted for an approximately 8-fold increase in RBA, the overall RBA is lower for ligands with the tertiary amine than without it due to the decrease in log *P*_*C*_. For example, RBA of ligand 16 is nearly 8-fold higher than that of ligand 15 that contains a piperidine substituent at the side chain terminus of ligand 16. Therefore, the role of the tertiary amine can be found from antiestrogenic properties by neutralizing Asp351 [39], rather than increased binding affinity by forming the salt-bridge.

Inspection of the 73 available ligand-bound hERα crystal structures indicates that a phenolic group primary forms a potent hydrogen bond with Glu353, and an additional hydroxyl group can form a hydrogen bond with Thr347 or His524 depending upon geometry of the ligand. The computational result of the symmetric compound diethylstilbestrol (DES) showed that hydrophobic contact with residues surrounding Glu353 (Leu349, Ala350, Leu387, Leu391, Phe404) is higher than that with residues around His524 (Met343, Met421, Ile424, His524, Leu525) by 0.13 which corresponds to 0.50 orders of magnitude in RBA. In conjunction with a strong hydrogen bond with Glu353, that contributes 0.52 orders of magnitude higher in RBA than that with His524, these hydrophobic interactions account for the binding mode of structurally diverse ligands.

**Fig 5:**
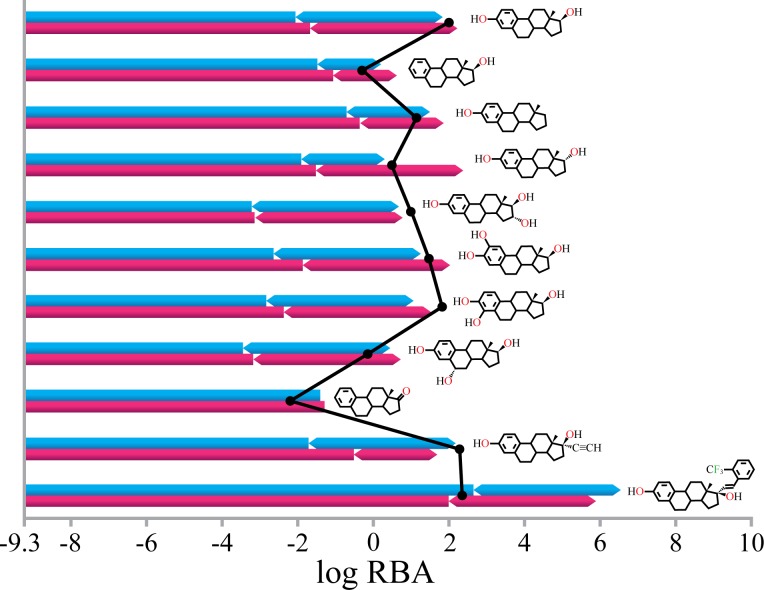
Prediction of RBA of 17β-estradiol derivatives based on protein-ligand complex structures from crystal structure modification (blue bars) or molecular docking (red bars). RBA is described by contributions from hydrophobic contacts (rectangle bars) and hydrogen bonds (hexagon bars). Black dots represent the experimental log RBA.

Introduction or elimination of a functional group in a ligand induces changes in steric and electronic properties that modify protein-ligand complex structure and bind affinity. The prediction results for E2 derivatives (Fig 5) showed that the 3D-QSAR can successfully explain the RBA variation according to the structural modification given the correct protein-ligand complex structure. Crystal structures of E2, 16α-hydroxyl E2, and 17α- trifluoromethylphenylvinyl E2 (PDB IDs: 1ERE, 3Q95, and 2P15) and conformations generated manually by adding or removing small functional groups to the crystal structures were used on the basis of an assumption that the rigid fused multi-ring moiety of E2 binds in a conserved manner. Modification of 3- or 17-hydoxyl groups resulted in loss of a hydrogen bond with Glu353 or His524, which accounts for 2.21 and 1.69 orders of magnitude decrease in RBA, respectively, but also an increase in hydrophobicity around C3 and C17. Due to the hydrophilic (carboxylate of Glu353 and guanidinium of Arg394) and hydrophobic (Met343, His524, and Leu525) environments of the C3 and C17, respectively, an increase in log *P*_*C*_ around C17 was more dominant than around C3. Introduction of polar groups, such as hydroxyl and ketone, decreased binding affinity by reducing hydrophobic contacts. E2 C2, 4, 6α, or 16α hydroxylation decreased log *P*_*C*_ by from 0.15 to 0.36 corresponding to from 0.58 to 1.39 orders of magnitude in RBA. RBA of 4-hydroxy E2 was underestimated by 7-fold due to the hydrogen bond with amide carbonyl of Leu346, which was not considered in this study. Introduction of a hydrophobic group at C17α position induced a less than 3-fold increase in RBA by extended hydrophobic contacts [27, 40]. The 17α-trifluoromethylphenylvinyl group formed extensive hydrophobic contacts with Phe404, Met343, Val418, Ile424, and Leu428, which increased log *P*_*C*_ by 1.22 corresponding to over 5000-fold increase in RBA. However, the observed RBA was comparable with E2 with 17α-etinyl group that increases log *P*_*C*_ by 0.08 corresponding to 2-fold increase in RBA. Possible explanation of this discrepancy between estimated and observed binding affinities is remodeling of H7 into an extended conformation for the accommodation of the bulky trifluoromethylphenylvinyl group [41] that likely destabilizes the protein structure. Comparison of these results with the prediction results based on docked conformations showed that the misidentification of hydrogen bonds (17α E2) and overestimation of log *P*_*C*_ (ethinyl E2), due to the incorrect protein-ligand complex structure, resulted in significant prediction error.

## Conclusion

In this study, we have developed a framework for quantitative analysis of binding affinity and mode of structurally diverse ERα agonists and antagonists based on 3D protein-ligand interaction description combined with the 3D-QSAR approach. Six interaction features including a salt-bridge with Asp351, three hydrogen bonds with Glu353, His524, and Thr347, the number of internal hydrogen bonds, and hydrophobic contacts were selected as key features for ERα binding. The hydrophobicity density field was introduced to account for stabilization of protein-ligand complex through hydrophobic contacts. The 3D-QSAR combined with molecular docking indicates that the dynamic character of the LBP allows accommodation and stable binding of structurally diverse ligands, and proper representation of protein flexibility is critical for reasonable description of binding affinity and mode of a diversity of ligands. Our results provide a rational explanation and accurate prediction of binding affinity and mode to *ER*α that may be applicable to other nuclear receptors.

## Supporting Information

S1 Fig Geometric criteria used to identify interactions between protein (*P*) and ligand (*L*). Hydrogen bonds are identified by distance (*d*) and angle (*θ*) between hydrogen bond donor and acceptor. (A) Hydrogen bonds of hydroxyl group from ligand with NH (donor) or N (acceptor) in His524. (B) Hydrogen bonds between hydroxyl group from ligand (donor) and hydrogen bond acceptor X including Glu353 and Thr347. (C) Salt-bridges between tertiary amine and Asp351 are identified by distance between the amine and carboxylate of Asp351. (D) Arrangement of phenyl group at the A-ring region is checked by distance from *m*_*P*_ to *m*_*L*_ and interplanar angle (*θ*). *m* is the ring mid-point, and *n* is normal to the plane of ring. The interactions were defined in binary terms, 0 or 1, representing forms or does not form, respectively. Parameter values are shown in the figure.
(TIF)

S2 Fig Selection of proper docked conformations of a ligand based on geometric relationship with His524. Docking experiments for the ligand were performed using three ERα structures (closed, moved back, and open. See article main text for detail) selected by structural similarity between a co-crystalized ligand and the ligand. Length of the ligand (*L*) and hydrogen bonds with Glu353 (*HB1*) and His524 (*HB2*) were considered.
(TIF)

S1 Table Hydrophobic residues in the ligand binding pocket used to estimate hydrophobic contact. (PDF)

S2 Table Estimated and observed relative binding affinities with protein-ligand interaction features. (PDF)

S1 File 73 ligand structures for model development. (SDF)

S2 File External validation set from EDKB. (SDF)
