# Supplementary figures and images for: Structure-Based Understanding of Binding Affinity and Mode of Estrogen Receptor α Agonists and Antagonists
Source: PLoS One. 2017 Jan 6;12(1):e0169607. doi: 10.1371/journal.pone.0169607 (PMC5218732; doi:10.1371/journal.pone.0169607)

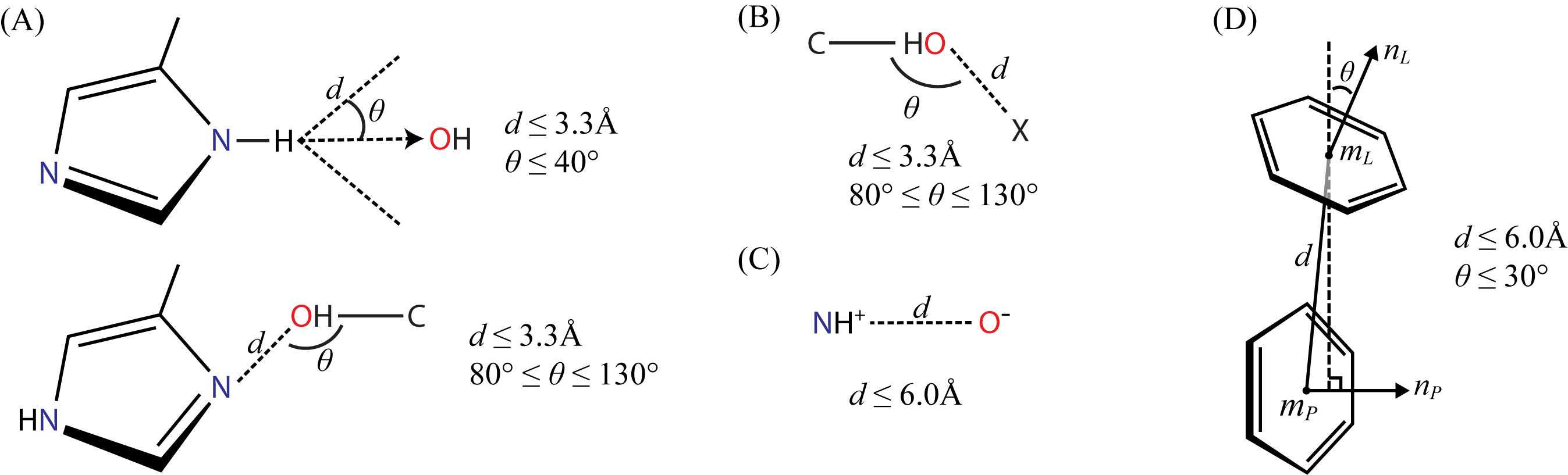

Supplement: S1 Fig — Hydrogen bonds are identified by distance (d) and angle (θ) between hydrogen bond donor and acceptor. (A) Hydrogen bonds of hydroxyl group from ligand with NH (donor) or N (acceptor) in His524. (B) Hydrogen bonds between hydroxyl group from ligand (donor) and hydrogen bond acceptor X including Glu353 and Thr347. (C) Salt-bridges between tertiary amine and Asp351 are identified by distance between the amine and carboxylate of Asp351. (D) Arrangement of phenyl group at the A-ring region is checked by distance from mP to mL and interplanar angle (θ). m is the ring mid-point, and n is normal to the plane of ring. The interactions were defined in binary terms, 0 or 1, representing forms or does not form, respectively. Parameter values are shown in the figure. (TIF) [file pone.0169607.s001.tif]

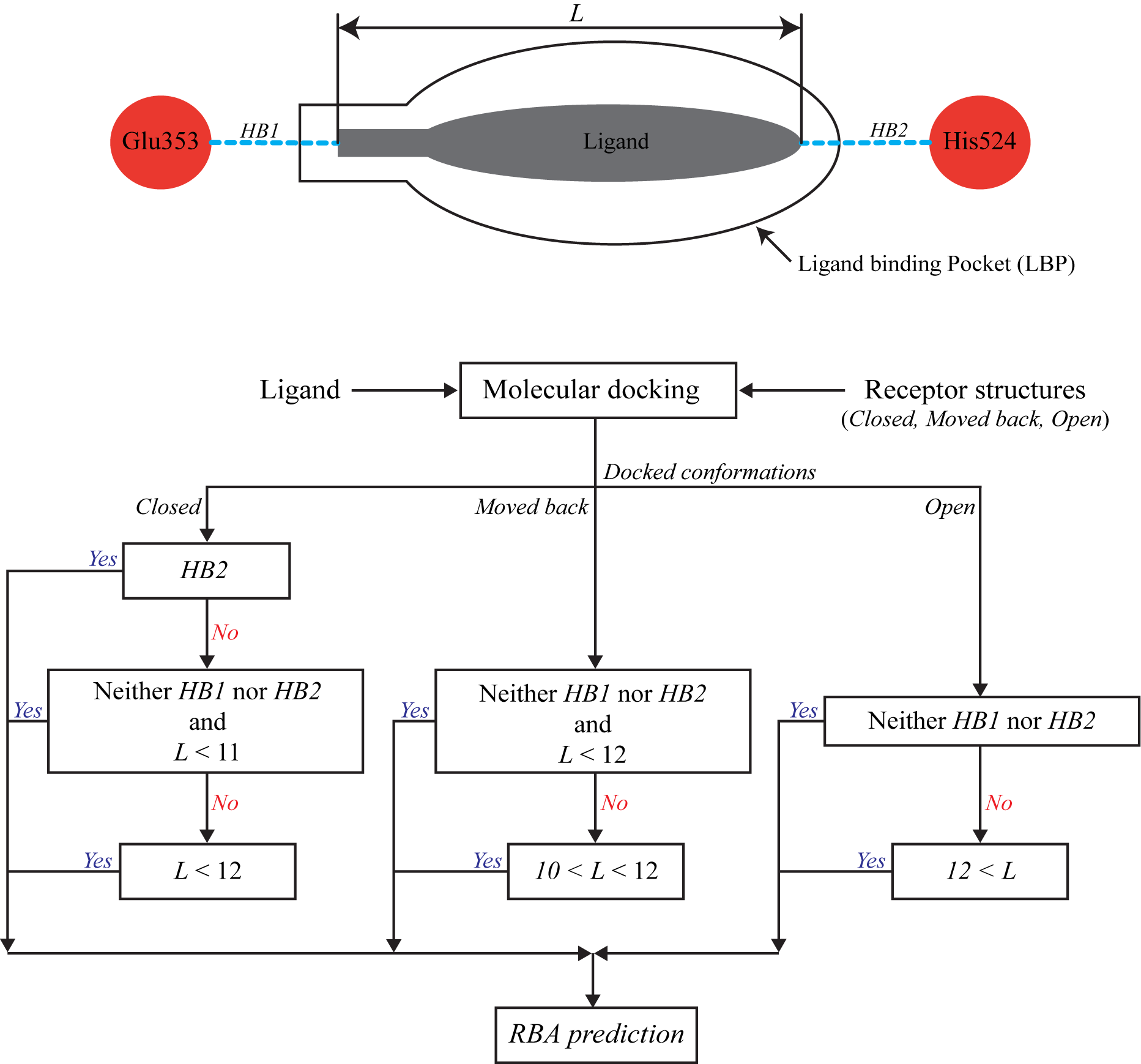

Supplement: S2 Fig — Docking experiments for the ligand were performed using three ERα structures (closed, moved back, and open. See article main text for detail) selected by structural similarity between a co-crystalized ligand and the ligand. Length of the ligand (L) and hydrogen bonds with Glu353 (HB1) and His524 (HB2) were considered. (TIF) [file pone.0169607.s002.tif]
