# Supplementary material for: Structure-Based Understanding of Binding Affinity and Mode of Estrogen Receptor α Agonists and Antagonists
Source: PLoS One. 2017 Jan 6;12(1):e0169607. doi: 10.1371/journal.pone.0169607 (PMC5218732; doi:10.1371/journal.pone.0169607)
Supplement: S1 Table — (PDF) [file pone.0169607.s003.pdf]

Amino acid	Residue Number	Atom
Met	343, 388, 421, 528	Cβ, Cγ, Sδ, Cε
Leu	346, 349,384,387, 391, 428, 525	Cβ, Cγ, Cδ1, Cδ2
Ala	350	Cβ
Val	418	Cβ, Cγ1, Cγ2
Phe	404	Cβ, Cγ, Cδ1, Cδ2, Cε1, Cε2, Cζ
Ile	424	Cβ, Cγ1, Cγ2, Cδ
His	524	Cβ, Cγ, CD2
