# Supplementary material for: Structure-Based Understanding of Binding Affinity and Mode of Estrogen Receptor α Agonists and Antagonists
Source: PLoS One. 2017 Jan 6;12(1):e0169607. doi: 10.1371/journal.pone.0169607 (PMC5218732; doi:10.1371/journal.pone.0169607)
Supplement: S2 Table — (PDF) [file pone.0169607.s004.pdf]

PDB	Receptor type	Ligand Name	Id	Fingerprint[b] FPs 1, 2, 3, 4, 5	FP6	Log RBA Exp	Cal
1ERE	closed	17β-Estradiol (E2)	1	0, 1, 1, 0, 0	1.85	2.00	1.71
1G50					2.05		2.47
1GWR					1.94		2.06
1PCG					1.98		2.21
1QKU					1.93		1.99
2OCF					1.86		1.72
2YJA					2.01		2.32
4PXM					1.95		2.08
3ERT	moved back	4-Hydroxytamoxifen	2	1, 1, 0, 0, 0	2.20	2.24	2.28
4Q50	open				1.99		1.47
1R5K	open	(2E)-3-{4-[(1Z)-1,2-Diphenyl-1-buten-1-yl]phenyl}acrylic acid	3	1, 0, 0, 0, 0	2.05	0.27	-0.49
5AAV	moved back				2.32		0.55
1SJ0	closed	(2S,3R)-3-(4-hydroxyphenyl)-2-[4-(2-piperidin-1-ylethoxy)phenyl]-2,3-dihydro-1,4-benzoxathiin-6-ol	4	1, 1, 1, 0, 0	1.74	2.29	2.20
1UOM	closed	(1R)-2-phenyl-1-[4-(2-piperidin-1-ylethoxy)phenyl]-3,4-dihydro-1H-isoquinolin-6-ol	5	1, 1, 0, 0, 0	1.96	1.56	1.36
1X7E	closed	2-[5-hydroxy-2-(4-hydroxyphenyl)-1-benzofuran-7-yl]ethanenitrile	6	0, 1, 1, 0, 0	1.24	-0.25	-0.66
1X7R	closed	Genistein	7	0, 1, 1, 0, 1	1.22	-0.36	-0.13
1XP1	closed	(2S,3R)-2-[4-[2-[(3R,4R)-3,4-dimethylpyrrolidin-1-yl]ethoxy]phenyl]-3-(4-hydroxyphenyl)-2,3-dihydro-1,4-benzoxathiin-6-ol	8	1, 1, 1, 0, 0	1.80	3.11	2.44
1XP6	closed	(2S,3R)-2-[4-[2-[(3S,4S)-3,4-dimethylpyrrolidin-1-yl]ethoxy]phenyl]-3-(4-hydroxyphenyl)-2,3-dihydro-1,4-benzoxathiin-6-ol	9	1, 1, 1, 0, 0	1.88	3.21	2.74
1XP9	closed	(2S,3R)-3-(4-hydroxyphenyl)-2-[4-[(2S)-2-pyrrolidin-1-ylpropoxy]phenyl]-2,3-dihydro-1,4-benzoxathiin-6-ol	10	1, 1, 1, 0, 0	1.86	2.70	2.66
1XPC	closed	(2S,3R)-3-(4-hydroxyphenyl)-2-[4-[(2R)-2-pyrrolidin-1-ylpropoxy]phenyl]-2,3-dihydro-1,4-benzoxathiin-6-ol	11	1, 1, 1, 0, 0	1.86	2.58	2.64
1XQC	moved back	(1R)-1-{4-[(9aS)-octahydro-2H-pyrido[1,2-a]pyrazin-2-yl]phenyl}-2-phenyl-1,2,3,4-tetrahydroisoquinolin-6-ol	12	1, 1, 0, 0, 0	2.00	1.18	1.52
1YIM	closed	(2R,3R,4S)-3-(4-hydroxyphenyl)-4-methyl-2-[4-(2-pyrrolidin-1-ylethoxy)phenyl]-3,4-dihydro-2H-chromen-6-ol	13	1, 1, 1, 0, 0	1.87	2.64	2.71
1YIN	closed	(2R,3R,4S)-5-fluoro-3-(4-hydroxyphenyl)-4-methyl-2-[4-(2-piperidin-1-ylethoxy)phenyl]-3,4-dihydro-2H-chromen-6-ol	14	1, 1, 1, 0, 0	2.01	2.91	3.25
2IOG	open	N-[(2R)-4-(4-hydroxyphenyl)butan-2-yl]-2-[2-phenyl-6-(2-piperidin-1-ylethoxy)-1H-indol-3-yl]ethanamide	15	1, 1, 0, 0, 0	2.01	1.91	1.54
2IOK	open	N-[(2R)-4-(4-hydroxyphenyl)butan-2-yl]-2-(2-phenyl-1H-indol-3-yl)ethanamide	16	0, 1, 0, 0, 0	2.44	2.81	2.28

PDB	Receptor type	Ligand Name	Id	Fingerprint[b] FPs 1, 2, 3, 4, 5	FP6	Log RBA Exp	Cal
2OUZ	moved back	(5R,6S)-6-phenyl-5-[4-(2-pyrrolidin-1-ylethoxy)phenyl]-5,6,7,8-tetrahydronaphthalen-2-ol	17	1, 1, 0, 0, 0	2.14	2.21	2.07
2QXS	closed	Raloxifene	18	1, 1, 1, 0, 0	1.95	2.12	3.00
3ERD	closed	Diethylstilbestrol	19	0, 1, 1, 0, 0	2.16	2.60	2.89
3Q95	closed	Estriol	20	0, 1, 1, 0, 0	1.57	0.99	0.60
3UU7	closed	Bisphenol A	21	0, 1, 0, 0, 0	1.27	-2.11	-2.24
3UUA	closed	Bisphenol AF	22	0, 1, 0, 1, 0	1.40	-0.11	-0.23
3UUC	closed	Bisphenol C	23	0, 1, 0, 1, 0	1.60	0.42	0.54
4MG5	closed	Kepone	24	0, 0, 0, 0, 0	1.86	-1.89	-2.15
4MG8	closed	α-Zearalanol	25	0, 1, 1, 0, 1	1.67	1.48	1.63
4MG9	closed	Butyl paraben	26	0, 1, 0, 0, 0	1.25	-3.07	-2.30
4MGA	closed	4-(2,4,4-trimethylpentan-2-yl)phenol	27	0, 1, 0, 0, 0	1.46	-1.82	-1.49
4MGC	closed	Bis(2,4-dihydroxyphenyl)methanone	28	0, 1, 1, 0, 2	0.80	-1.03	-1.13
4MGD	closed	HPTE	29	0, 1, 0, 1, 0	1.57	-0.60	0.41
4TUZ	closed	α-Zearalenol	30	0, 1, 1, 0, 1	1.62	1.63	1.44
4TV1	closed	Propyl 4-hydroxybenzoate	31	0, 1, 0, 0, 0	0.99	-3.22	-3.32
1ERR	closed	Raloxifene	32	1, 1, 1, 0, 0	1.89	NA	2.78
1GWQ	closed	2-(4-hydroxyphenyl)-1-benzothiophen-6-ol	33	0, 1, 1, 0, 0	1.45	NA	0.14
1ZKY	closed	4-[(1S,2S,5S,6S,9S)-5-(hydroxymethyl)-6,8,9-trimethyl-3-oxabicyclo[3.3.1]non-7-en-2-yl]phenol	34	0, 1, 1, 0, 0	1.64	NA	0.88
2AYR	open	6-(4-methylsulfonylphenyl)-5-[4-(2-piperidin-1-ylethoxy)phenoxy]naphthalen-2-ol	35	1, 1, 0, 0, 0	1.60	NA	-0.04
2B1V	closed	4-[(1s,2s,5s)-5-(hydroxymethyl)-8-methyl-3-oxabicyclo[3.3.1]non-7-en-2-yl]phenol	36	0, 1, 1, 0, 0	1.51	NA	0.40
2B1Z	moved back	17-Methyl-17-Alpha-Dihydroequilenin	37	0, 0, 0, 0, 0	2.15	NA	-1.04
2FAI	closed	4-[(1R,2S,5S,9R)-5-(hydroxymethyl)-8,9-dimethyl-3-oxabicyclo[3.3.1]nonan-2-yl]phenol	38	0, 1, 1, 0, 0	1.44	NA	0.13
2G44	moved back	4-[(1s,2r,5s)-4,4,8-Trimethyl-3-Oxabicyclo[3.3.1]non-7-En-2-Yl]phenol	39	0, 1, 0, 0, 0	1.74	NA	-0.41
2P15	closed	(17β)-17-{(E)-2-[2-(trifluoromethyl)phenyl]vinyl}estra-1(10),2,4-triene-3,17-diol	40	0, 1, 1, 0, 0	3.07	NA	6.41
2POG	closed	(3AS,4R,9BR)-4-(4-hydroxyphenyl)-1,2,3,3A,4,9B-hexahydrocyclopenta[C]chromen-9ol	41	1, 1, 0, 0, 0	1.78	NA	0.65
2Q70	closed	(3AS,4R,9BR)-2,2-difluoro-4-(4-hydroxyphenyl)-1,2,3,3A,4,9B-hexahydrocyclopenta[C]chromen-8-ol	42	1, 1, 0, 0, 0	1.78	NA	0.68

PDB	Receptor type	Ligand		Fingerprint[b]		Log RBA	
		Name	Id	FPs 1, 2, 3, 4, 5	FP6	Exp	Cal
2R6W	closed	[6-hydroxy-2-(4-hydroxyphenyl)-1-benzothiophen-3-yl]{4-[2-(4-methylpiperidin-1-yl)ethoxy]phenyl}methanone	43	1, 1, 1, 0, 0	2.04	NA	3.38
2R6Y	closed	[6-hydroxy-2-(4-hydroxyphenyl)-1-benzothiophen-3-yl][4-(2-pyrrolidin-1-ylethoxy)phenyl]methanone	44	1, 1, 1, 0, 0	1.94	NA	2.96
3DT3	closed	5-(4-hydroxyphenoxy)-6-(3-hydroxyphenyl)-7-methyl-naphthalen-2-ol	45	1, 1, 0, 0, 0	2.30	NA	2.67
4IU7	closed	4-[2-ethyl-7-(trifluoromethyl)indazol-3-yl]benzene-1,3-diol	46	0, 1, 0, 0, 0	0.81	NA	-4.01
4IUI	open	4-[1-butyl-7-(trifluoromethyl)indazol-3-yl]benzene-1,3-diol	47	0, 1, 0, 0, 1	1.36	NA	-1.27
4IV2	open	4-[1-(2-methylpropyl)-7-(trifluoromethyl)indazol-3-yl]benzene-1,3-diol	48	0, 0, 0, 0, 1	1.72	NA	-2.09
4IV4	moved back	4-[2-(2-methylpropyl)-7-(trifluoromethyl)indazol-3-yl]benzene-1,3-diol	49	0, 1, 0, 0, 0	1.77	NA	-0.31
4IVW	closed	4-[2-benzyl-7-(trifluoromethyl)-2H-indazol-3-yl]benzene-1,3-diol	50	0, 1, 0, 0, 0	2.15	NA	1.16
4IVY	moved back	4-[1-but-3-enyl-7-(trifluoromethyl)indazol-3-yl]benzene-1,3-diol	51	0, 1, 0, 0, 1	1.68	NA	-0.03
4IW6	moved back	4-[2-but-3-enyl-7-(trifluoromethyl)indazol-3-yl]benzene-1,3-diol	52	0, 1, 0, 0, 0	1.86	NA	0.03
4IW8	open	4-[1-(3-methylbut-2-enyl)-7-(trifluoromethyl)indazol-3-yl]benzene-1,3-diol	53	0, 1, 0, 0, 1	1.71	NA	0.09
4IWC	open	4,4'-thiene-2,5-diylbis(3-methylphenol)	54	0, 1, 0, 0, 0	2.18	NA	1.28
4IWF	closed	2-chloro-3'-fluoro-3-[(E)-(hydroxyimino)methyl]biphenyl-4,4'-diol	55	0, 1, 1, 0, 1	1.37	NA	0.47
4MG6	closed	benzyl butyl benzene-1,2-dicarboxylate	56	0, 0, 0, 0, 0	2.02	NA	-1.55
4MG7	closed	Ferutinine	57	0, 0, 0, 0, 0	2.41	NA	-0.03
4MGB	closed	4,4'-propane-2,2-diylbis(2,6-dichlorophenol)	58	0, 0, 0, 0, 0	1.52	NA	-3.49
4PP6	closed	Resveratrol	59	0, 1, 1, 0, 0	1.13	NA	-1.07
4PPP	closed	Fluoro-resveratrol	60	0, 1, 1, 0, 0	0.95	NA	-1.77
4PPS	closed	(1S,3aR,5R,7aS)-5-(4-hydroxyphenyl)-7a-methyloctahydro-1H-inden-1-ol	61	0, 1, 1, 0, 0	1.56	NA	0.56
4ZN9	open	Cyclohexa-2,5-dien-1-yl (1S,2R,4S)-5,6-bis(4-hydroxyphenyl)-7-oxabicyclo[2.2.1]hept-5-ene-2-sulfonate	62	0, 1, 0, 0, 0	1.75	NA	-0.36
5AAU	closed	3-(1-(4-Chlorophenyl)-3,4-dihydro-1H-pyrido(3,4-b)indol-2(9H)-yl)propanoic acid	63	0, 0, 1, 0, 0	0.68	NA	-5.02
5AK2	moved back	(E)-3-[4-[[3-(4-fluoranyl-2-methyl-phenyl)-7-oxidanyl-2-oxidanylidene-chromen-4-yl]methyl]phenyl]prop-2-enoic acid	64	1, 1, 0, 0, 0	1.89	NA	1.10

[a]NA: Not available

[b]FP1: salt-bridge with Asp351, FP2-FP4: hydrogen bond with Glu353, His524, and Thr347, respectively, FP5: number of internal hydrogen bonds, FP6: hydrophobic contact
